# Effects of Sodium Butyrate Supplementation in Milk on the Growth Performance and Intestinal Microbiota of Preweaning Holstein Calves

**DOI:** 10.3390/ani13132069

**Published:** 2023-06-22

**Authors:** Donglin Wu, Zhanhe Zhang, Kai Shao, Xing Wang, Fudong Huang, Jingwei Qi, Yizong Duan, Yang Jia, Ming Xu

**Affiliations:** 1 College of Animal Science, Inner Mongolia Agricultural University, Hohhot 010018, China; wdl2017@emails.imau.edu.cn (D.W.); nddky@emails.imau.edu.cn (Z.Z.); 15184732117@163.com (K.S.); fudong@emails.imau.edu.cn (F.H.); jiayang@imau.edu.cn (Y.J.); 2 Inner Mongolia Herbivorous Livestock Feed Engineering and Technology Research Center, Hohhot 010018, China; 3 Shazhou Dairy Co., Ltd., Ulanqab 013750, China

**Keywords:** calf feeding, sodium butyrate, milk, intestinal microbiota, growth performance

## Abstract

**Simple Summary:**

Sodium butyrate is an important nutritional additive for livestock animals and its potential for improving growth performance is of great interest. The present study reported the optimal sodium butyrate supplementation level in milk for preweaning dairy calves and the effects of sodium butyrate supplementation in milk on the growth performance and intestinal microbiota of calves around the newborn stage and provided evidence of the effects of sodium butyrate supplementation in milk for dairy calf feeding.

**Abstract:**

The aim of the present study was to investigate the effects of sodium butyrate (SB) supplementation on the growth and intestinal microbiota of preweaning dairy calves. Eighty newborn Holstein calves (56 female and 24 male) were randomly allocated to four treatment groups with 20 calves each (14 female and 6 male). The suckling milk for the four treatments was supplemented with 0, 4.4, 8.8, or 17.6 g/d SB. During the 6-week experiment, dry matter intake was recorded daily, body weight was measured weekly, and rectal fecal samples were collected in the 2nd week. The V3–V4 hypervariable regions of the microbial 16S rRNA were amplified and then sequenced. SB supplementation elevated average daily gains (ADGs) in the first and second weeks. The optimal SB supplementation level for the whole preweaning period was 8.78 g/d, as revealed by analyzing the whole preweaning period ADG using second-order polynomial regression (quadratic) equations. The alpha diversity (Shannon diversity index), beta diversity, core phyla and genera, and function of the intestinal microbiota were affected by SB supplementation. In addition, the Shannon diversity index and core phyla and genera of the intestinal microbiota were correlated with calf growth-related indices. Overall, SB supplementation in suckling milk improved the growth performance and intestinal microbiota development of dairy calves in a quadratic manner, and regression analysis indicated an optimal supplementation level of 8.78 g/d.

## 1. Introduction

Calf feeding and management for optimal growth are increasingly receiving attention from farmers. Improved growth is related to a better health status in preweaning calves and thus to dairy farm interests and weaning success [1,2]. In addition, a growing body of evidence has shown that the milk yield of dairy cows is affected by their growth and health in the suckling period [3,4]. Promoting the development of the intestinal microbiota may be a promising way to address these issues because postnatal levels of intestinal microbes fluctuate until maturity and mediate the growth rate and long-term health outcome of neonates [5]. Short-chain fatty acids are synthesized in the gastrointestinal tract by anaerobic microbes during fermentation of food substrates, and butyrate in particular is the main fuel for ruminal and intestinal epithelial cells [6]. Immediately after birth, the production of endogenous butyrate in neonates’ gastrointestinal tracts is relatively low and it gradually increases with age as the intestinal microbiota matures [6,7]. During critical temporal windows of neonatal development, butyrate plays a role in the maturation of the host’s endocrine, metabolic, and immune systems [8]. Therefore, butyrate is an important nutritional additive for livestock and its potential for improving the growth performance of newborn calves is of great interest [6,9].

Exogenous butyrate supplements, such as sodium butyrate (SB) and tributyrin, have been introduced in the starter used for preweaned calves [10,11,12] and liquid feeds (milk, milk replacer, or a mixture of milk and milk replacer) [13,14,15,16]. Cow milk contains naturally free butyrate and triacylglycerol-esterified butyrate in milk fat, which can be digested and released by pregastric lipase and pancreatic lipase in preweaned calves [17,18]. For structural and functional development of the gastrointestinal tract, stimulation with free butyrate together with milk-borne hormones and growth factors has been considered sufficient, although the free butyrate concentration in cow milk is relatively low [6]. In addition, for feeding, milk seems to have an advantage over the milk replacer in terms of effects on preweaning calf growth performance [19,20]. These factors may explain why no study has evaluated SB supplementation in milk using milk as a single source of liquid feed in dairy calf feeding. Moreover, supplementation within the starter for neonatal calves might not meet host needs because of the low starter intake of calves. Butyrate supplementation in milk might be more reasonable and feasible for neonatal calves, even a daily 45 g supplementation in liquid feed (mixture of milk and milk replacer) [14].

There have been relatively few studies evaluating the effects of butyrate supplementation on the intestinal microbiota. Protected SB supplemented in milk replacer has been reported to change the abundance of short-chain fatty acid-producing and health-associated bacteria in the cecum microbiota of preweaned calves [13]. Tributyrin supplemented in milk replacer also stimulated colonization by short-chain fatty acid-producing bacteria in the jejunum and ileum of preweaned calves [16]. However, there is also a lack of evidence for the effect of SB supplementation in milk on the intestinal microbiota of calves, which is important since many dairy farms use milk for calf feeding. The two mentioned studies provided evidence of the long-term effects of butyrate supplementation on the intestinal microbiota in preweaned calves [13,16]. To our knowledge, there is a lack of evidence for the effect of butyrate supplementation on the intestinal microbiota of calves near the newborn stage in calf feeding. Studies conducted during the process of microbial colonization of the calves, not at the end of colonization, might be more important, since the process of microbial colonization has high plasticity and variability, especially in preweaning calves [21]. It is known that short-term SB supplementation has a more obvious effect on growth performance near the newborn stage [10,11], which might contribute to the promoting effects of butyrate on the intestinal microbiota; however, this needs clarification in calf feeding.

Therefore, based on previous studies, the objectives of this study were (1) to test whether suckling milk needs exogenous SB supplementation when dairy calves are fed milk only as liquid feed and (2) to evaluate the relationship between exogenous SB supplementation and the intestinal microbiota near the newborn stage of neonatal dairy calves receiving milk.

## 2. Materials and Methods

### 2.1. Animals and Experimental Procedure

This study procedure was reviewed and approved by the Institutional Animal Care and Use Committee at Inner Mongolia Agricultural University (protocol No. 2020099) and was performed in accordance with the guiding principles of the Humane Treatment of Laboratory Animals (HTLA Pub. Chapter 2–6, revised 2006 in China).

The study, which began on 23 August 2020 and ended on 11 October 2020, was conducted at Shazhou Dairy Co., Ltd. (Liangcheng County, Ulanqab, China; latitude 40.53° N and longitude 112.49° E) and all calves used in this study were provided by this farm. The location had a mid-temperate continental monsoon climate, an average relative humidity of 30.78 ± 30.67% (mean ± S.D.), and an average temperature of 18.42 ± 6.85 °C. The relative humidity and ambient temperature were measured daily in the morning (06:00 to 08:00), afternoon (13:00 to 15:00), and evening (18:00 to 20:00), and these data were measured every 10 min by Hobo Pro Series Temp probes (Onset Computer Co., Pocasset, MA, USA) throughout the experimental period; the probes were hung approximately 1.0 m above the ground. All calves (removed from their dams immediately after birth) were selected from healthy multiparous Holstein dams (with 3–4 parities) and housed individually in outdoor calf hutches (1.35 m in height, 1.5 m in width, and 2.2 m in depth) with dry sand for bedding; the hutches were cleaned manually at 10:00 every day. The calves underwent a clinical examination prior to initiation of the experiment and were monitored throughout the experimental period. A pasteurized colostrum (IgG > 50.0 g/L; measured by a colostrum densimeter; Kruuse, Langeskov, Denmark) was fed to each calf at a volume corresponding to 10% of the calf’s birth weight within the first 1 h of life, and 2 L of pasteurized colostrum was fed to each calf after 6 h; thereafter, pasteurized milk was given to the calves. In this study, normal whole milk was used and not the waste milk from the farm. Pasteurized milk was given in an individual open bucket to each calf, and the bucket was cleaned after every milk feeding and dripped dry before the next meal. Pasteurized milk (4.4 L/d for d 4–10, and 8.8 L/d for d 11–45) was equally divided and fed to the preweaning calves over two meals throughout the day, at 05:00 and 17:00. In the weaning transition period, the calves were divided into groups of ten and housed indoors (1.5 m in height, 10 m in width, and 15 m in length) with dry sand for bedding, and a compatible playground (1.5 m in height, 15 m in width, and 15 m in length) with dry sand was also provided. The bedding was manually cleaned every 3 d. A mixture with equal amounts of milk and milk replacer was provided at d 46 and thereafter in a step-down manner (also divided into two meals a day; 8 L/d for d 46–50, 6 L/d for d 51–55, and 4 L/d for d 56–58); the calves were completely weaned after the morning feeding on d 59 (2 L). The temperatures of both the milk and mixture of milk and milk replacer were 36 to 37 °C. In the preweaning period, calves were provided starter feed and water (15.92 ± 2.11 °C) ad libitum daily in two buckets that were maintained at the calf hutches, and no hay was provided to the calves. The commercial pelleted starter feed was purchased from Tianjin Jiuzhou Dadi Feed Co., Ltd. (Tianjin, China) and fed to the calves from d 3. In the weaning transition period, the same starter was provided ad libitum in the trough for calves.

Eighty healthy Holstein female (56 calves) and male (24 calves) calves (body weight, BW; 41.72 ± 2.81 kg) aged 2–4 d (2.88 ± 0.45 d) were randomly allocated to one of four treatments (20 calves per treatment; 14 females and 6 males) based on age and BW. The treatments consisted of milk with different amounts of SB supplementation for calves: (1) without SB (0 g/d), CON; (2) with a low level of SB (4.4 g/d), LSB; (3) with a medium level of SB (8.8 g/d), MSB; and (4) with a high level of SB (17.6 g/d), HSB. Butyrate is available as a sodium, potassium, magnesium, or calcium salt, and the advantage of salts over free acid is that they are generally odorless and easier to handle in the feed manufacturing process because of their solid and less volatile state [6]. Therefore, SB, as the source of exogenous for butyrate supplementation, was added in the milk of neonatal calves. The SB product (Jiabaoyu; ≥98% SB; Jinan Degao Agriculture and Animal Husbandry Technology Co., Ltd., Jinan, China) used was a raw powder. The SB supplementation level was determined in our pre-experiment (Supplemental Table S1). Since no promotion effect was detected when preweaning calves were fed 17.6 g/d SB or more in milk, this level was selected as the highest supplementation level, and two intermediate values of 0 and 17.6 g/d, i.e., 4.4 and 8.8 g/d, were selected and applied in the present study. Specifically, the SB was provided via two daily milk feedings, at 2.2, 4.4, and 8.8 g per feeding for the LSB, MSB, and HSB treatments, respectively. For SB supplementation, SB was manually stir-mixed into individual open buckets sufficiently for each calf in the SB treatment groups prior to each feeding.

### 2.2. Feed Intake, Growth-Related Indices, and Sample Collection for GIM

The feed intake of each calf was determined daily at 19:00; the residual amount was recorded (except during heavy rains), and the dry matter intake (DMI) of the calves was calculated. The postnatal calves were weighed on d 3 (initial weight) and thereafter every week until d 45 (final weight) and, based on the average daily gain (ADG), the feed efficiency (FE) for each week of each calf was calculated as DMI/ADG. The height at the withers, body length, and hearth girth of each calf were measured on d 3 (initial) and d 45 (final), and the gains in these parameters (the height at the withers, body length, and hearth girth) were calculated. Representative samples of each feed sample (200 g) were obtained weekly and kept frozen at −20 °C until being pooled together for analysis. The feed samples were analyzed according to the corresponding reference method [22], the feed samples were analyzed: dry matter (DM) (method 930.15), crude protein (method 976.05) and ether extract (method 920.39). Neutral detergent fiber and acid detergent fiber levels were determined according to previously described methods [23] with a fiber analyzer (Ankom 220; Ankom Co., Macedon, NY, USA). The neutral detergent fiber content was analyzed with heat-stable α-amylase and sodium sulfite. Both neutral detergent fiber and acid detergent fiber levels are expressed inclusive of residual ash. The starch content was analyzed using a modified glucoamylase-based method [24]. Starch was hydrolyzed to free D-glucose and the glucose concentration was then measured. The observed chemical compositions of the feed are shown in Supplemental Table S2. In the present study, 30 mL of milk (without SB supplementation) was sampled daily in the preweaning period at both the morning (15 mL) and afternoon (15 mL) calf feeding times, and an equivolume mixture was prepared and frozen at −20 °C until the time of analysis. The samples were thawed and thoroughly homogenized before being used for assays. The concentration of free butyrate was determined using gas chromatography–mass spectrometry as previously described [25]; the average concentration was 14.96 ± 2.88 mg/100 mL (ranging from 10 to 20 mg/100 mL) in the six-week sampling period.

On the last day of the 2nd week (d 17) of the experiment, the ten calves (seven females and three males, at the same age and initial BW) of each group were selected for intestinal microbiota sample collection. Fecal samples were collected from the rectum by hand using sterile gloves (to ensure no contact with the environment and avoid microbial contamination), transferred into sterile and pyrogen-free centrifuge tubes, immediately frozen in liquid nitrogen and stored at −80 °C until further analysis.

### 2.3. DNA Extraction, PCR Amplification, and 16S rRNA Sequencing

Total fecal sample DNA was extracted from fecal samples using the E.Z.N.A.^®^ Soil DNA Kit (Omega Bio-Tek, Norcross, GE, USA) according to the manufacturer’s instructions. The quality of the extracted DNA was checked by 1% agarose gel electrophoresis, and the DNA concentration and purity were determined with a NanoDrop 2000 UV–vis spectrophotometer (Thermo Scientific, Waltham, MA, USA). The V3–V4 hypervariable regions of the microbial 16S rRNA genes were amplified and then sequenced with the Illumina MiSeq platform (Majorbio BioPham Technology, Shanghai, China) using the primers 338F (5′-ACTCCTACGGGAGGCAGCA-3′) and 806R (5′-GGACTACHVGGGTWTCTAAT-3′). PCR was performed in triplicate under the following conditions: initial denaturation at 95 °C for 3 min, followed by 29 cycles of 95 °C for 30 s, 55 °C for 30 s, and 72 °C for 45 s and a final extension at 72 °C for 10 min. The PCR mixture included 2 μL of 5 × PrimeSTAR buffer (4 μL), dNTPs (2.5 mM), 2 μL of forward primer (5 μM), 0.8 μL of reverse primer (5 μM), 0.8 μL of PrimeSTAR hot-start DNA polymerase, and 20 ng of template DNA. Two-percent agarose gels were used to detect the PCR products, which were then purified using a DNA purification kit (Axygen Biosciences, Union City, CA, USA). The raw 16S rRNA sequences were demultiplexed, quality-filtered by fastp version 0.20.0 and merged by FLASH version 1.2.7 using previously reported criteria. Operational taxonomic units (OTUs) with a 97% similarity cut off were clustered using UPARSE (version 7.0; http://drive5.com/uparse/, accessed on 15 March 2022) and chimeric sequences were identified and removed [26]. The taxonomy of each OTU was assigned by classifying its representative sequence using the RDP Classifier algorithm (http://rdp.cme.msu.edu/, accessed on 12 November 2021) against the Silva 16S rRNA database (SSU123; https://www.arb-silva.de/, accessed on 12 November 2021) with a confidence threshold of 70% [27].

### 2.4. Microbial Data Processing

Alpha diversity was evaluated with the Shannon diversity index, Chao1 estimator, and Shannon index-based evenness. Beta diversity (between sample diversity) was analyzed using the weighted UniFrac distance and unweighted UniFrac distance, followed by Adonis, and visualized using nonmetric multidimensional scaling (NMDS). Adonis is a nonparametric method used to test the differences in community structure among populations. Venn diagrams were used to illustrate the core microbiota at the phylum, genus, and OTU levels. In addition, core bacteria at the phylum and genus levels were filtered out as those accounting for ≥0.1% among all four groups. Pearson’s correlation coefficients were determined between data generated from the intestinal microbiota (Shannon, Chao1, Shannon index-based evenness, and relative abundance of biomarkers from the taxonomic analysis of the microbiota at the phylum and genus levels) and the calves’ feed intake and growth-related indices. Functional profiles of microbial communities were predicted using phylogenetic investigation of communities by reconstruction of unobserved states (PICRUSt), as it is used to predict the association between phylogeny and function to estimate the metabolomics functional profile of a microbial community using 16S rRNA amplicon sequences [28]; the results of the level 1 and 2 KEGG pathways are presented in this paper.

### 2.5. Statistical Analysis

Data on growth-related indices (BW, ADG, DMI, FE, and gain of withers height, body length, and hearth girth) were analyzed using one-way ANOVA in the General Linear Models procedure (SAS; version 9.2; SAS Institute Inc., Cary, NC, USA), and differences in means were adjusted by the Tukey—Kramer test. Orthogonal polynomial linear and quadratic contrasts were used to examine treatment effects (SB supplementation levels). Alpha diversity indices (Shannon diversity index, Chao1 estimator, and Shannon index-based evenness), relative abundance of the microbiota at the phylum and genus levels, and relative abundance of the enriched KEGG pathways (levels 1 and 2) were analyzed in the same way as the calves’ growth-related indices. GraphPad Prism (version 8.0.2; GraphPad Software, Inc., San Diego, CA, USA) was used to prepare the figures, and Pearson’s correlation analysis and 2-tailed significance tests were performed in the software. In the present study, data were considered significant at *p* < 0.05.

## 3. Results

### 3.1. Growth-Related Indices

One female calf in the LSB group suffered from respiratory diseases and severe diarrhea at week 4, and the calf was removed from the data analysis of growth-related indices at week 4 and thereafter. During the whole period, we did not observe any refusal from calves for intake of milk with exogenous SB supplementation, indicating that the calf intake of the SB supplement was at the expected level. Growth-related indices are shown in Table 1 (for the whole period) and Supplemental Figure S1A–C (BW, DMI, and FE for each week). During the whole period, a difference in the DMI of calves among the four treatments was observed (*p* = 0.020), and the ADG increased quadratically with increasing SB supplementation level (*p* = 0.005). During the 1st, 2nd, 3rd, 4th, and 5th weeks, the ADG increased quadratically with increasing SB supplementation level (*p* < 0.05) and calves in the LSB, MSB, and HSB groups had a higher ADG than the CON group calves during the 1st and 2nd weeks (*p* < 0.05). There was no difference in the FE and gain of height at the withers, body length, or hearth girth for calves in the whole period (*p* > 0.05). In addition, the growth performance (BW and ADG) of the grouped feeding calves that were fed a mixture containing equal amounts of milk and milk replacer at d 46 and thereafter is shown in Supplemental Figure S1D,E for the 7th and 8th weeks (weaned at d 59 of age), which shows that there were no differences among the four groups (*p* > 0.05).

**Figure 1:**
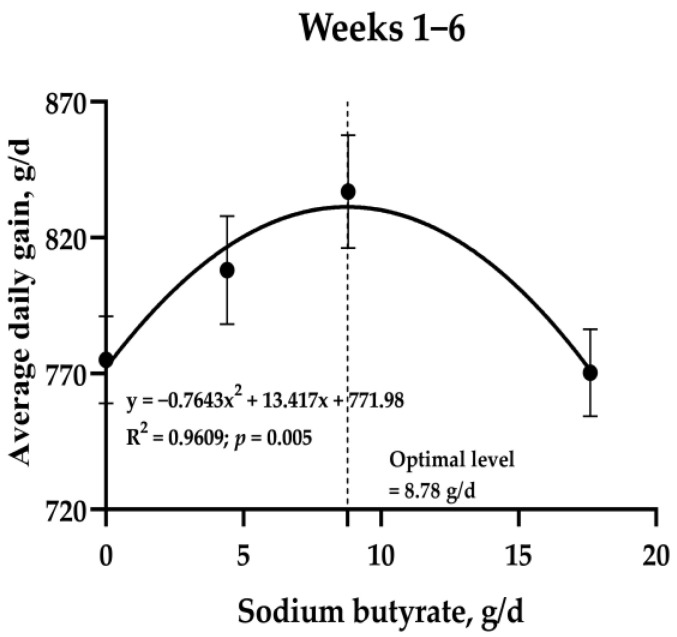
The optimal sodium butyrate supplementation level in milk for the whole period average daily gain was analyzed using second-order polynomial regression (quadratic) equations for preweaning calves. *n* = 20 (*n* = 19 for the LSB group at week 4 and thereafter).

In addition, the results of second-order polynomial regression (quadratic) on ADG in the whole experimental period showed that the optimal SB supplementation level for the whole period was 8.78 g/d (*p* = 0.005; R^2^ = 0.96, Figure 1). We also performed an analysis using second-order polynomial regression (quadratic) on ADG for each week, and the optimal SB supplementation levels were 12.33, 11.24, 7.74, 6.71, and 6.59 g/d for the 1st, 2nd, 3rd, 4th, and 5th weeks, respectively. The 6th week was not used in the analysis because there were no differences (as tested by orthogonal polynomial linear and quadratic contrasts) in ADG, as shown in Table 1. The obtained optimal SB supplementation level for each week was then analyzed using a regression analysis, and interestingly, the level decreased as the number of weeks increased (*p* = 0.016, R^2^ = 0.89; Figure S2).

### 3.2. Diversity of the Intestinal Microbiota

**Figure 2:**
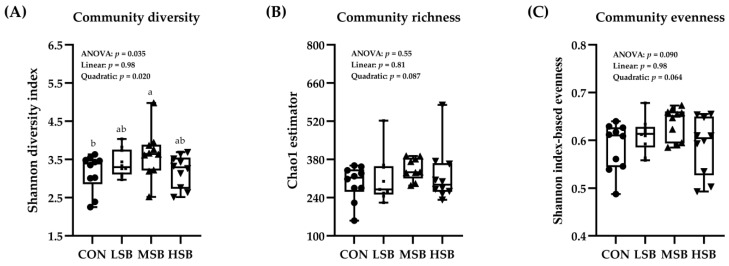
Effects of sodium butyrate supplementation on the alpha diversity of the intestinal microbiota in preweaning calves. Alpha diversity was evaluated with the Shannon diversity index (**A**), Chao1 richness estimator (**B**), and Shannon index-based evenness (**C**). CON = treatment consisted of milk without SB supplementation for calves (0 g/d), LSB = treatment consisted of milk with a low level of SB supplementation for calves (4.4 g/d), MSB = treatment consisted of milk with a medium level of SB supplementation for calves (8.8 g/d), and HSB = treatment consisted of milk with a high level of SB supplementation for calves (17.6 g/d); Data were considered significant at *p* < 0.05; *n* = 10 for each group; ^a–b^ Means without a common superscript differ significantly (*p* < 0.05).

To further investigate the changes in intestinal microbiota communities after milk supplementation with SB, 16S rRNA sequencing was conducted. A total of 2,194,527 clean reads were obtained from rectal samples (Supplemental Table S3), and the Good’s coverage indices in the four groups were all greater than 99%, indicating that most of the bacteria present in the samples were identified and that the sequencing depth was adequate for the community analysis. The rarefaction curves tended to be flat (Figure S3), suggesting that a reasonable number of individual samples were taken from all four groups, since the rarefaction curves were used to estimate the completeness of microbial community sampling. Thus, the data were sufficient for the analysis of microbial communities. The alpha diversity index results are shown in Figure 2A–C. The microbiota diversity (Shannon diversity index, Figure 2A) changed quadratically with increasing SB supplementation level (*p* < 0.05), and the MSB group had a higher Shannon index than the CON and HSB groups (*p* < 0.05). In addition, there were no differences in richness (Chao1 richness estimator) or evenness (Shannon index-based evenness) among the four groups (*p* > 0.05, Figure 2B,C). The NMDS plot of beta diversity indices, based on community membership as measured by the weighted UniFrac distance and unweighted UniFrac distance followed by the Adonis test, showed that there were large distances among the intestinal microbes for each group, indicating low similarity (stress = 0.128; R^2^ = 0.139, *p* = 0.027; Figure S4A), and the same results were observed for the unweighted UniFrac distance (stress = 0.116; R^2^ = 0.134, *p* = 0.001; Figure S4B). The stress value is used to check the quality of the NMDS analysis results. When the stress is less than 0.20, it can be represented by a two-dimensional point graph of NMDS, which has certain explanatory importance.

More OTUs were detected in the LSB, MSB, and HSB groups than in the CON group according to the Venn diagram (Supplemental Figure S5A), and more bacteria were identified at the genus (Supplemental Figure S5B) and phylum (Supplemental Figure S5C) levels, with more microorganisms in the MSB group. For example, 366 (21.34%) shared common OTUs among the four groups were observed at the OTU level, and the number of unique microorganisms in the CON, LSB, MSB, and HSB groups was 31 (1.81%), 195 (11.37%), 423 (24.66%), and 38 (2.22%), respectively (a greater number of unique microorganisms suggests a greater number of microorganisms overall).

### 3.3. Composition of the Intestinal Microbiota

**Figure 3:**
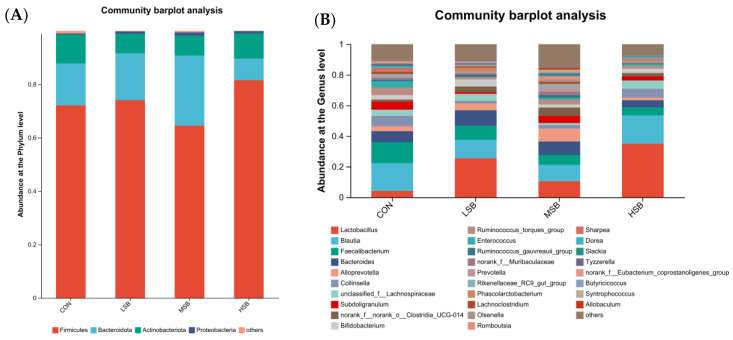
Effects of sodium butyrate supplementation in milk on the intestinal microbiota (relative abundance) at the phylum and genus levels in preweaning calves. Community bar diagram at the phylum (**A**) and genus (**B**) levels, showing taxa with relative abundance ≥0.1%. CON = treatment consisted of milk without SB supplementation for calves (0 g/d), LSB = treatment consisted of milk with a low level of SB supplementation for calves (4.4 g/d), MSB = treatment consisted of milk with a medium level of SB supplementation for calves (8.8 g/d), and HSB = treatment consisted of milk with a high level of SB supplementation for calves (17.6 g/d). *n* = 10 for each group.

A taxonomic analysis of the intestinal microbiota (relative abundance) at the phylum (Figure 3A) and genus (Figure 3B) levels was performed and is shown as a community bar diagram and microbial community pie-plot. Core and dominant bacteria at the phylum and genus levels were filtered out as those accounting for ≥0.1% among all four groups. Four bacterial phyla (*Firmicutes*, *Bacteroidota*, *Actinobacteriota*, *Proteobacteria*) (Figure 3A) and twenty-eight bacterial genera (*Lactobacillus*, *Blautia*, *Faecalibacterium*, *Bacteroides*, *Alloprevotella*, *Collinsella*, *unclassified_f__Lachnospiraceae*, *Subdoligranulum*, *norank_f__norank_o__Clostridia_UCG-014*, *Bifidobacterium*, *Ruminococcus_torques_group*, *Enterococcus*, *Ruminococcus_gnavus_group*, *norank_f__Muribaculaceae*, *Prevotella*, *Rikenellaceae_RC9_gut_group*, *Phascolarctobacterium*, *Lachnoclostridium*, *Olsenella*, *Romboutsia*, *Dorea*, *Slackia*, *Tyzzerella*, *norank_f__Eubacterium_coprostanoligenes_group*, *Butyricicoccus*, *Syntrophococcus*, and *Allobaculum*) (Figure 3B) were detected. At the phylum level, the order from high to low in terms of relative abundance was *Firmicutes*, *Bacteroidota*, *Actinobacteriota*, and *Proteobacteria* in the CON, LSB, and MSB groups and *Firmicutes*, *Actinobacteriota*, *Bacteroidota*, and *Proteobacteria* in the HSB group. At the genus level, among the detected core and dominant twenty-eight bacterial genera, from high to low, the top ten relative abundances in the four groups were *Lactobacillus*, *Blautia*, *Bacteroides*, *Faecalibacterium*, *norank_f__norank_o__Clostridia_UCG-014*, *norank_f__Muribaculaceae*, *Alloprevotella*, *Rikenellaceae_RC9_gut_group*, *Collinsella*, and *unclassified_f__Lachnospiraceae*.

For the four bacterial phyla and twenty-eight bacterial genera mentioned above, the difference (*p* < 0.05) in relative abundance at the phylum and genus levels among the four groups after SB supplementation (tested by ANOVA and the Tukey—Kramer test and orthogonal polynomial linear and quadratic contrasts) is shown in Table 2, and the results with no difference (*p* > 0.05) are presented in Supplemental Table S4. There was a difference in the relative abundance of one bacterial phylum (*Bacteroidota*) and ten bacterial genera (*Lactobacillus*, *Rikenellaceae_RC9_gut_group*, *norank_f__Eubacterium_coprostanoligenes_group*, *Lachnoclostridium*, *Olsenella*, *norank_f__Muribaculaceae*, *Lachnospiraceae_NK4A136_group*, *Ruminococcus*, *Romboutsia*, and *Erysipelotrichaceae_UCG-003*) (*p* < 0.05; Table 2). The relative abundance of the phylum *Bacteroidota* increased quadratically with increasing SB supplementation level (*p* = 0.038). The LSB and HSB groups had a higher abundance of *Lactobacillus* than the CON and MSB groups (*p* < 0.05). The MSB group had a higher abundance of *norank_f__Muribaculaceae* and *Rikenellaceae_RC9_gut_group* than the LSB and HSB groups (*p* < 0.05). The relative abundance of *norank_f__Eubacterium_coprostanoligenes_group* decreased linearly with increasing SB supplementation level (*p* = 0.016), and the CON group had a higher abundance of this genus than the other three groups (*p* < 0.05). A higher abundance of *Lachnoclostridium* was detected in the CON and MSB groups than in the HSB group (*p* < 0.05). A higher abundance of *Ruminococcus* was detected in the MSB group than in the CON and HSB groups (*p* < 0.05). The relative abundance of *Olsenella* increasing linearly with increasing SB supplementation level (*p* = 0.048). The relative abundances of *Romboutsia* (*p* = 0.038) and *Lachnospiraceae_NK4A136_group* (*p* = 0.004) increased quadratically with increasing SB supplementation level. The MSB group had a higher relative abundance of *Romboutsia* than the other three groups (*p* < 0.05), and the MSB group had a higher relative abundance of *Lachnospiraceae_NK4A136_group* than the CON group (*p* < 0.05). A higher relative abundance of *Erysipelotrichaceae_UCG-003* was detected in the HSB group than in the other three groups (*p* < 0.05). In addition, *Allobaculum* was detected in only some samples (less than half of samples) in all four groups, and the genus was not examined in this study.

### 3.4. Function of the Intestinal Microbiota

Six level 1 KEGG pathways (relative abundance, %) were identified, shown in Table 3, and forty-six level 2 KEGG pathways (relative abundance, %) were identified and are shown in Supplemental Table S5. At level 1, there were no changes among the four groups in environmental information processing and metabolism (*p* > 0.05). The pathway involved in cellular processes was enriched in the CON and MSB groups compared with the HSB group (*p* < 0.05) and the pathway involved in genetic information processing was enriched in the CON and MSB groups compared with the LSB and HSB groups (*p* < 0.05). Pathways involved in human diseases were enriched in the LSB and HSB groups compared with the CON group (*p* < 0.05), and pathways involved in organismal systems were enriched in the LSB and HSB groups compared with the CON and MSB groups (*p* < 0.05). Analysis of level 2 KEGG pathways (Supplemental Table S5) showed that health-related pathways included drug resistance: antineoplastic, cancers: overview, immune diseases and cancers: specific types, which were generally enriched in the LSB and HSB groups compared with the CON and MSB groups (*p* < 0.05).

### 3.5. Correlation of Growth-Related Indices and the Intestinal Microbiota

**Figure 4:**
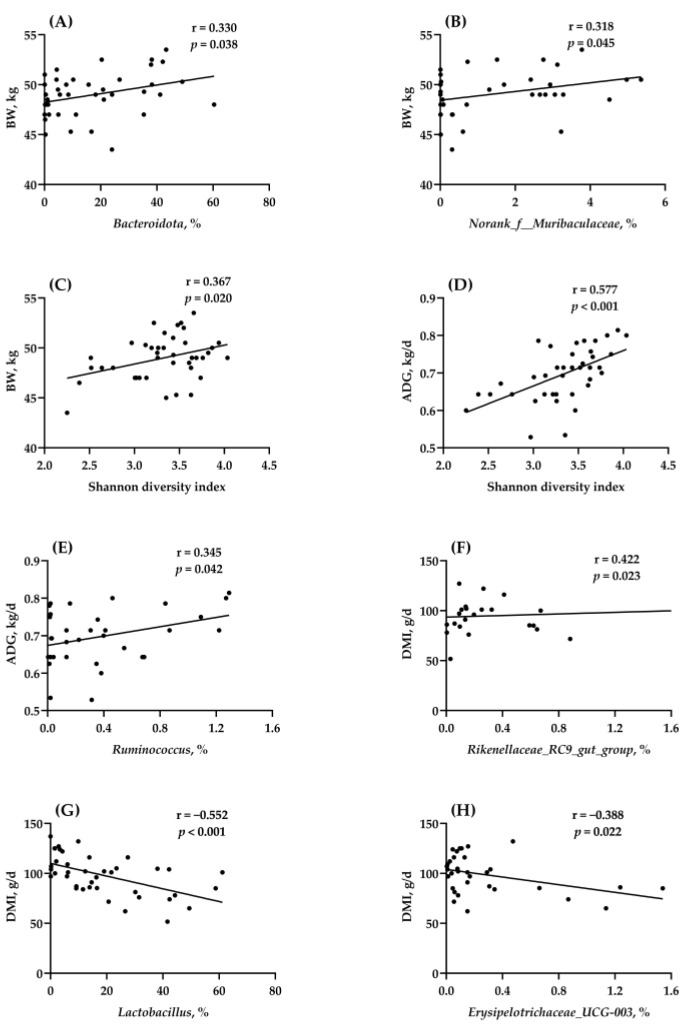
Relationships between the growth-related indices and the relative abundance of the intestinal microbiota at the phylum and genus levels. The correlation (r) and *p* value were tested using Pearson’s rank correlation coefficient and 2–tailed significance tests. BW: body weight; ADG: average daily gain; DMI: dry matter intake. (**A**) BW and *Bacteroidota* (phylum level), (**B**) BW and *norank_f__Muribaculaceae* (genus level), (**C**) BW and Shannon diversity index, (**D**) ADG and Shannon diversity index, (**E**) ADG and *Ruminococcus* (genus level), (**F**) DMI and *Rikenellaceae_RC9_gut_group* (genus level), (**G**) DMI and *Lactobacillus* (genus level), and (**H**) DMI and *Erysipelotrichaceae_UCG-003* (genus level). Data were considered significant at *p* < 0.05; *n* = 40.

The growth performance results of calves in the 2nd week were selected for correlation analysis, including the DMI and BW at the end of the 2nd week (d 17) and ADG and FE in the 2nd week (d 11–17). Some bacterial genera had zero abundance in some individual samples, and those with zero abundance were removed in the correlation analysis. The correlation between the intestinal microbiota and growth-related indices of preweaning calves determined from the differences in the present study (Table 1 and Supplemental Figure S1A–C) is presented in Figure 4 (correlated results; *p* < 0.05) and Supplemental Table S6 (uncorrelated results; *p* > 0.05). As shown in Figure 4A–C, the abundances of *Bacteroidota* (r = 0.330, *p* = 0.038) and *norank_f__Muribaculaceae* (r = 0.318, *p* = 0.045) and the Shannon diversity index (r = 0.318, *p* = 0.045) were correlated positively with the BW at the end of the 2nd week in preweaning calves. The Shannon diversity index (Figure 4D; r = 0.577, *p* < 0.001) and *Ruminococcus* abundance (Figure 4E; r = 0.345, *p* = 0.042) were correlated positively with ADG in the 2nd week in preweaning calves. The abundances of *Rikenellaceae_RC9_gut_group* (Figure 4F; r = 0.422, *p* = 0.023), *Lactobacillus* (Figure 4G; r = −0.552, *p* < 0.001), and *Erysipelotrichaceae_UCG-003* (Figure 4H; r = −0.388, *p* = 0.022) were negatively correlated with DMI at the end of the 2nd week (d 17) in preweaning calves. In addition, there was no correlation (*p* > 0.05) between the intestinal microbiota and FE in the 2nd week in preweaning calves (Supplemental Table S6).

## 4. Discussion

In the present study, one of the objectives was to test whether exogenous butyrate supplementation is needed when dairy calves are fed milk only as liquid feed. The results showed that dairy calves exhibited a higher BW and ADG in the preweaning period, especially in the newborn stage (2nd week), when milk was supplemented with exogenous butyrate. The structural and functional development of the gastrointestinal tract stimulated by butyrate, as free butyrate and triacylglycerol-esterified butyrate in milk fat, together with milk-borne hormones and growth factors, have been considered sufficient [6,17]. This result is supported by a recent study showing that tributyrin supplemented in pasteurized waste milk played a role in reducing oxidative stress and inflammation in preweaning dairy calves [15]. Butyrate is released in the abomasum from milk fat via the action of pregastric lipase in calves [17] but the secretion and potency of pregastric lipase is highest near the newborn stage and decreases with increasing calf age [18]. Endogenous production of butyrate from the fermentation of milk by the microbiota and pancreatic lipase in the gastrointestinal tract of calves increased with calf age [6,29]. Therefore, it is of interest to clarify the relationship between pregastric and pancreatic lipase action and microbiota action on butyrate released from milk fat with advancing calf age in future studies.

The other objective was to evaluate the relationship between exogenous butyrate supplementation and the intestinal microbiota around the newborn stage of neonatal dairy calves receiving milk feeding and it was determined that SB supplementation promoted intestinal microbiota development in calves in the 2nd week of life. This result filled the gap left by studies aimed at butyrate supplementation on the intestinal microbiota near weaning. In the 2nd week, we reported that exogenous SB provided in milk increased the intestinal microbiota diversity (Shannon diversity index) in a quadratic manner and the effects were especially evident at a moderate supplementation level. It is thought that high diversity within an ecosystem promotes the stability, productivity, and function of the system [30]. A higher growth rate linked to increased intestinal microbial community diversity has also been described in human infants [31]. In the present study, the diversity indices were positively and significantly correlated with the growth of calves (BW and ADG).

For core and dominant bacterial phyla and genera in the present study, we reported that the phylum *Bacteroidota* and genera *norank_f__Muribaculaceae*, *Rikenellaceae_RC9_gut_group*, and *Ruminococcus* contributed to the DMI, BW, or ADG, which we observed to be increased by SB treatments. The phylum *Bacteroidota*, formerly called *Bacteroidetes*, is considered a key player in the healthy state, sophisticated homeostasis, and immunomodulation [32] and plays a vital trophic role in polysaccharide catabolism, carbohydrate fermentation, and amino acid and protein utilization [33,34]. Thus, this evidence indicated that the phylum *Bacteroidota* was related to BW in this study. The genus *norank_f__Muribaculaceae*, which was enriched in the MSB group and correlated positively with BW, might contribute to the regulation of animal lipid metabolism. This genus has also been reported to alleviate colitis in mice [35] and it was found that *norank_f__Muribaculaceae* was positively correlated with milk yield in cows [36]. *Muribaculaceae*, historically called *S24-7*, belongs to the phylum *Bacteroidetes*. This family is composed of trophic guilds that specialize in the degradation of polysaccharides, such as host glycans, plant glycans, and α-glucans [37]. *Muribaculaceae* species are also equipped with fermentation pathways to produce acetate, propionate, and succinate in carbohydrate degradation [38]. *Rikenellaceae_RC9_gut_group* was enriched mostly in the MSB group, and this bacterial genus is known for its digestion of fiber and was found to be positively correlated with ADG in cattle [39,40]. In addition, the family *Rikenellaceae* can participate in the production of short-chain fatty acids and in the scavenging of hydrogen in the gastrointestinal tract, thus lowering methanogenesis rates [41]. All of these studies reinforce the notion that this bacterial genus improves energy storage for calves, consistent with our results showing that this bacterial genus was correlated positively with DMI. In addition, some potentially beneficial bacterial genera—*Olsenella*, *Lachnospiraceae_NK4A136_group*, and *Romboutsia*—were enriched by SB supplementation, which might reveal the effect of SB supplementation on the growth performance of calves. For example, *Lachnospiraceae_NK4A136_group* has been linked to a higher digestibility of fiber and production of short-chain fatty acids, resulting in a higher ADG in cattle [42].

In the present study, the potentially pathogenic bacterium *Erysipelotrichaceae_UCG-003* was also detected and this genus was negatively correlated with DMI. *Erysipelotrichaceae_UCG-003* has been reported to be associated with intestinal dysfunction as well as the induction of inflammatory bowel diseases and disorders of bile acid metabolism [43,44]. *Erysipelotrichaceae* has also been reported to serve as a biomarker of low feed efficiency in lactating dairy cows [45]. *Norank_f__Eubacterium_coprostanoligenes_group*, known as a cholesterol-reducing bacterial genus, has long been known to be involved in bile acid metabolism and linked to a decreased in the blood cholesterol content [46], as well as being known for its anti-inflammatory role in the intestine [47]. We observed that the relative abundance of the bacterium decreased linearly with the SB supplementation level, suggesting that the disturbance in lipid metabolism and inflammation with SB supplementation was relatively low. In addition, a study of meat ducks also indicated that a decline in the abundance of *Eubacterium_coprostanoligenes_group* was accompanied by an increase in ADG [48].

The intestinal microbiota reacted to the proper dosage of SB supplementation. The reaction was observed at the phylum level; the phylum *Bacteroidota* reacted in a quadratic way, and a high supplementation level reduced the abundance of the phylum. The phylum *Bacteroidota* plays a role in the healthy state, sophisticated homeostasis, immunomodulation, and trophic function [32,33,34]. Therefore, a high SB supplementation level will be detrimental to calves. At the genus level, *Lactobacillus* reacted with the proper dosage of SB supplementation, since we observed that the genus was over-enriched (four- to nine-fold) in the LBS and HSB groups. *Lactobacillus* is a genus of probiotic bacteria with numerous beneficial effects on host metabolism and health [5]. However, over-enrichment of *Lactobacillus* in the LBS and HSB groups had a negative impact on the DMI of calves, which might be explained by the overgrowth of *Lactobacillus*, leading to harmful acidotic conditions for host metabolism [49]. Functional analysis also revealed that the intestinal microbiota reacted to proper SB supplementation levels, as the disease-related genes were enriched in the LSB and HSB groups, as shown using PICRUSt analysis of the level 1 and 2 KEGG pathways. The results of this study were supported by some evidence that supplementation with excess butyrate reduced the proportion of beneficial bacteria in the intestines and was toxic to the metabolic function of mice [50,51].

Calves fed milk supplemented with butyrate can consume more butyrate near the newborn stage when the host microbiota is immature [6,7] and this cannot be achieved by using starter supplemented with butyrate since calves have low starter intake during this period. In addition, we did not observe any refusal from calves to consume milk with exogenous SB supplementation even at 17.6 g/d (2 g/L in milk), which ensured that SB intake occurred at the expected level; this is also attributed to the properties of SB itself, since it dissolves easily in milk, unlike tributyrin (extreme low solubility), which is a kind of fat and might adhere to feeding tools [52]. Therefore, supplementation in milk could be a reasonable and effective method for ensuring SB intake during dairy calf feeding and might be more advantageous than starter supplementation.

## 5. Conclusions

In conclusion, the results from the present study indicated that suckling milk should be supplemented with SB for preweaning calves; the optimal supplementation amount is 8.78 g/d. Moreover, SB supplementation in milk, especially near the newborn stage, is important because supplementation can improve intestinal microbiota development and growth performance. This study provided evidence of the benefits of suckling milk supplemented with SB and the relationship between SB supplementation and the intestinal microbiota during dairy calf feeding.

## Figures and Tables

**Table 1:** Effects of sodium butyrate supplementation in milk on feed intake and growth-related indices in preweaning calves.

Items	Supplementation Level, g/d				SEM	*p*-Value		
	0	4.4	8.8	17.6		ANOVA	Linear	Quadratic
Initial BW, kg	39.83	39.70	39.81	39.84	0.55	0.99	0.93	0.91
Final BW, kg	72.02	73.75	75.00	72.72	0.75	0.096	0.013	0.014
DMI, g/d	142.55 ^ab^	121.34 ^bc^	147.64 ^a^	115.03 ^c^	8.66	0.020	0.73	0.39
ADG, g/d								
Week 1	554.33 ^b^	619.1 ^a^	673.54 ^a^	654.63 ^a^	15.83	0.002	0.001	0.011
Week 2	655.84 ^b^	709.09 ^a^	727.45 ^a^	710.03 ^a^	13.16	0.039	0.008	0.025
Week 3	910.18	941.98	945.86	888.57	14.67	0.17	0.099	0.045
Week 4	890.6	907.98	915.00	847.31	14.47	0.079	0.15	0.044
Week 5	927.82	950.42	946.83	889.08	13.23	0.067	0.16	0.045
Week 6	931.93	935.71	932.54	915.79	9.01	0.62	0.73	0.49
In the whole period	775.03 ^b^	808.09 ^ab^	836.97 ^a^	770.37 ^b^	13.93	0.037	0.009	0.005
FE	0.162	0.138	0.150	0.135	0.009	0.12	0.31	0.60
Gain of height at the withers, cm	10.25	10.44	10.47	9.64	0.58	0.43	0.42	0.23
Gain of body length, cm	13.15	13.74	13.35	13.29	0.39	0.76	0.53	0.51
Gain of hearth girth, cm	18.72	18.87	19.56	18.50	0.36	0.20	0.093	0.064

SEM = standard error of the means; *n* = 20 (*n* = 19 for the LSB group at week 4 and thereafter). BW: body weight; ADG: average daily gain; DMI: dry matter intake. FE: feed efficiency, calculated as DMI/ADG. ^a–c^ Means without a common superscript within a row differ significantly (*p* < 0.05).

**Table 2:** Effects of sodium butyrate supplementation in milk on the taxonomic analysis of the intestinal microbiota in preweaning calves at the phylum and genus levels.

Items	Supplementation Level, g/d				SEM	*p*-Value		
	0	4.4	8.8	17.6		ANOVA	Linear	Quadratic
Phylum level								
*Bacteroidota*	15.79	17.95	26.85	8.16	5.09	0.095	0.084	0.038
Genus level								
*Lactobacillus*	4.11 ^b^	25.16 ^a^	10.03 ^b^	34.95 ^a^	3.98	<0.001	0.47	0.70
*norank_f__Muribaculaceae*	1.284 ^ab^	0.996 ^b^	2.563 ^a^	0.667 ^b^	0.481	0.045	0.095	0.059
*Rikenellaceae_RC9_gut_group*	1.474 ^ab^	0.348 ^b^	2.301 ^a^	0.232 ^b^	0.494	0.015	0.38	0.223
*norank_f__Eubacterium_ coprostanoligenes_group*	1.497 ^a^	0.440 ^b^	0.240 ^b^	0.063 ^b^	0.323	0.015	0.016	0.082
*Lachnoclostridium*	1.511 ^a^	0.784 ^ab^	1.224 ^a^	0.221 ^b^	0.258	0.007	0.63	0.71
*Ruminococcus*	0.092 ^b^	0.446 ^ab^	0.644 ^a^	0.081 ^b^	0.062	<0.001	<0.001	<0.001
*Olsenella*	0.241	0.733	1.361	0.522	0.198	0.23	0.048	0.054
*Romboutsia*	0.229 ^b^	0.248 ^b^	1.940 ^a^	0.292 ^b^	0.260	0.042	0.036	0.038
*Lachnospiraceae_NK4A136_group*	0.028 ^b^	0.178 ^ab^	0.491 ^a^	0.124 ^ab^	0.055	0.013	0.003	0.004
*Erysipelotrichaceae_UCG-003*	0.110 ^b^	0.133 ^b^	0.089 ^b^	0.564 ^a^	0.058	0.005	0.39	0.067

SEM = standard error of the means; *n* = 10 for each group. ^a,b^ Means without a common superscript within a row differ significantly (*p* < 0.05).

**Table 3:** Enriched predicted level 1 KEGG pathways (relative abundance, %).

Items	Supplementation Level, g/d				SEM	*p*-Value		
	0	4.4	8.8	17.6		ANOVA	Linear	Quadratic
Cellular processes	5.38 ^a^	5.02 ^ab^	5.30 ^a^	4.65 ^b^	0.09	0.007	0.96	0.42
Environmental information processing	14.21	13.37	14.01	13.72	0.13	0.098	0.35	0.43
Genetic information processing	5.85 ^a^	6.50 ^b^	5.95 ^a^	6.66 ^b^	0.09	<0.001	0.65	0.72
Human diseases	2.52 ^b^	2.72 ^a^	2.63 ^ab^	2.68 ^a^	0.02	0.011	0.052	0.13
Metabolism	70.97	71.13	70.99	71.04	0.12	0.97	0.88	0.89
Organismal systems	1.070 ^b^	1.266 ^a^	1.131 ^b^	1.253 ^a^	0.020	<0.001	0.16	0.45

SEM = standard error of the means; *n* = 10 for each group. ^a,b^ Means without a common superscript within a row differ significantly (*p* < 0.05).

## Data Availability

The datasets that are applied and/or analyzed throughout study are available from the corresponding author upon reasonable request. In addition, the 16S rRNA amplicon sequencing data generated during the current study were submitted to NCBI under BioProject PRJNA904681.

## Supplementary Materials

The following supporting information can be downloaded at: https://www.mdpi.com/article/10.3390/ani13132069/s1, Table S1. The growth performance, health scores, and incidence of diarrhea in Holstein preweaning calves aged 36–50 d that were fed different levels of sodium butyrate in milk. Table S2. Ingredients and chemical compositions of the feed. Table S3. Information on the sequencing depth for 16S rRNA. Table S4. Effects of sodium butyrate supplementation on the taxonomic analysis of the intestinal microbiota at the phylum and genus levels in preweaning calves. Table S5. Effects of sodium butyrate supplementation on KEGG pathways (level 2) enriched in the intestinal microbiota in preweaning calves as analyzed using the PICRUSt program. Table S6. Relationship of growth-related indices of calves and indices of the intestinal microbiota. Figure S1. Growth performance of preweaning calves in each week and the body weight and average daily gain of calves at 46–59 d of age (weeks 7 and 8; weaning transition period). Figure S2. Result of regression analysis for the obtained optimal sodium butyrate supplementation level for each week. Figure S3. Rarefaction curves of the intestinal microbiota in preweaning calves. Figure S4. Beta diversity of the intestinal microbiota in preweaning calves. Figure S5. Venn diagram of the common and distinct taxa at the phylum, genus, and OTU levels among the four groups of preweaning calves.
