# Supplementary material for: Effects of Sodium Butyrate Supplementation in Milk on the Growth Performance and Intestinal Microbiota of Preweaning Holstein Calves
Source: Animals (Basel). 2023 Jun 22;13(13):2069. doi: 10.3390/ani13132069 (PMC10340040; doi:10.3390/ani13132069)
Supplement: Supplementary file 1 [file animals-13-02069-s001.zip › animals-2321140-supplementary.pdf]

Supplementary material – Tables and Figures

Supplemental Table S1. The growth performance, health scores, and incidence of diarrhea in Holstein preweaning calves aged 36–50 d that were fed different levels of sodium butyrate in milk.

Items	Sodium butyrate supplement level (Trt, g/L)				SEM	*p*-value					
	0 (0 g/d)	2 (17.6 g/d)	4 (35.2 g/d)	6 (52.8 g/d)		ANOVA	Linear	Quadratic	Trt	Day	Trt × Day
Initial BW, kg	59	59.08	60.6	58.25	2.57	0.93	0.64	0.59	0.84	<0.001	0.99
Final BW, kg	77.26	76.67	79.12	77.12	2.71	0.93	0.80	0.78			
ADG, g/d	1074.12	1034.31	1089.41	1109.8	56.66	0.81	0.92	0.76	–	–	–
DMI, g/d	382.04	335.28	351.6	402.64	23.8	0.20	0.64	0.59	0.13	<0.001	0.92
Feed efficiency	0.35	0.32	0.32	0.38	0.04	0.70	0.38	0.29	–	–	–
Ear score	1.07^{c}	1.22^{b}	1.37^{a}	1.21^{b}	0.04	<0.001	<0.001	0.001	<0.001	<0.001	0.21
Eye score	1.30	1.26	1.33	1.22	0.04	0.096	0.92	0.51	0.10	<0.001	0.42
Nasal discharge score	1.37	1.36	1.47	1.31	0.07	0.43	0.32	0.27	0.47	0.22	0.90
Fecal scores	2.32	2.16	2.43	2.44	0.07	0.099	0.49	0.18	0.066	<0.001	0.80
Incidence of diarrhea,%	14.76	9.23	21.79	18.3	5.4	0.13	0.22	0.51	0.12	0.031	0.97

SEM = standard error of the means; $n = 6$ for each group; means within row with the different lowercase letters superscripts are significantly different ($p < 0.05$). BW = body weight; ADG = average daily gain; DMI = dry matter intake; Feed efficiency = DMI/ADG. All the calves fed with 8.8 L/d whole milk. Ear scores were (1) normal, (2) ear flick or head shake, (3) slight unilateral droop, and (4) head tilt or bilateral droop. Eye scores were (1) normal, (2) small amount of ocular discharge, (3) a moderate amount of bilateral discharge, and (4) heavy ocular discharge. Nasal discharge scores were (1) normal serous discharge, (2) small amount of unilateral cloudy discharge, (3) bilateral or cloudy (or both) or excessive mucus discharge, and (4) copious bilateral mucopurulent discharge. Fecal scores were established as (1) normal, (2) semi-formed, pasty, or both, (3) loose but stays on top of bedding, and (4) watery or sifts through the bedding, or both.

Supplemental Table S2. Ingredients and chemical compositions of the feed.

Ingredient, of % DM[1]		Chemical analysis (DM, %)	
Corn	40.0	Nutrients of % DM	88.30
Wheat bran	8.0	Crude protein	23.61
Distiller's dried grains with soluble	5.0	Ether extract	5.73
Corn husk	10.0	Ash	8.37
Soybean meal	16.0	Neutral detergent fiber	21.67
Cotton meal	16.0	NFC[3]	40.62
Premix[2]	5.0	Acid detergent fiber	12.36
Total	100.0	Starch	35.21

[1] DM = dry matter.

[2] Composition of premix per kilogram: pantothenic acid 200 mg; niacin acid 400 mg; folic acid 20 mg; biotin 3 mg; I 10 mg; Se 10 mg; Co 10 mg; Fe 5,000 mg; Cu 1,000 mg; Zn 2,000 mg; Mn 2,000 mg; vitamin A 300,000 IU; vitamin D 150,000 IU; vitamin E 280,000 IU; vitamin K 80 mg; vitamin B1 30 mg; vitamin B2 100 mg; vitamin B6 60 mg; vitamin B12 0.4 mg.

[3] Non-fiber carbohydrate (NFC) is calculated as 100 − (crude protein + ether extract + ash + neutral detergent fiber).

Supplemental Table S3. Information on the sequencing depth for 16S rRNA genes.

Sample ID	Total read bases (bp)	Clean reads	Mean_length	Min_length	Max_length	Goods -coverage
WK2CON1	30345884	74557	407.015894	212	441	0.998935
WK2CON2	20109665	49582	405.583982	231	471	0.99899
WK2CON3	18967614	45632	415.664753	245	483	0.998401
WK2CON4	22795915	54724	416.561563	216	479	0.999323
WK2CON5	20370553	49205	413.993558	337	430	0.998217
WK2CON6	21289619	52162	408.144224	252	439	0.998254
WK2CON7	23175712	56608	409.40701	241	431	0.998668
WK2CON8	24187359	58408	414.110379	228	522	0.998788
WK2CON9	22119689	53923	410.208798	275	430	0.998594
WK2CON10	22480269	54197	414.788069	231	454	0.998136
WK2LSB1	29656798	71261	416.17151	212	464	0.996815
WK2LSB2	21961995	52794	415.994147	210	490	0.998892
WK2LSB3	21959993	52774	416.113863	265	431	0.999045
WK2LSB4	20711183	50618	409.166364	246	499	0.998689
WK2LSB5	21121475	50727	416.375402	246	464	0.998908
WK2LSB6	24341721	58380	416.953083	259	464	0.998128
WK2LSB7	19926977	47315	421.155595	212	442	0.998368
WK2LSB8	22531348	53918	417.881746	270	490	0.999028
WK2LSB9	23526633	56647	415.320017	239	477	0.998733
WK2LSB10	20685532	49316	419.448698	270	441	0.996611
WK2MSB1	21173912	51216	413.423774	225	441	0.998695
WK2MSB2	30884239	73674	419.201333	232	432	0.998639
WK2MSB3	25158768	60379	416.680766	327	431	0.998339
WK2MSB4	23123059	55781	414.532888	330	431	0.998672
WK2MSB5	20726880	50382	411.394546	216	431	0.99832
WK2MSB6	22468769	53537	419.68674	266	478	0.997783
WK2MSB7	20067903	47681	420.8784	216	441	0.995408
WK2MSB8	21296019	51186	416.051635	252	435	0.998154
WK2MSB9	24351273	58860	413.715138	270	490	0.998639
WK2MSB10	18934503	45164	419.238841	216	450	0.998356
WK2HSB1	30167901	73756	409.023008	230	464	0.998895
WK2HSB2	27378256	65386	418.717401	220	492	0.998393
WK2HSB3	22348917	53863	414.921505	228	446	0.998123
WK2HSB4	23899837	57578	415.086266	216	431	0.998777
WK2HSB5	19838684	48479	409.22222	216	430	0.998072
WK2HSB6	23605076	56533	417.54508	215	478	0.998894
WK2HSB7	16593022	40074	414.05954	238	432	0.998475
WK2HSB8	22620472	54219	417.205629	252	431	0.99814
WK2HSB9	22380013	53484	418.443142	301	465	0.996356
WK2HSB10	20632399	50547	408.182464	337	433	0.998558
Summation		**2194527**				
Average	22747895.9	54863.175	414.6817243	247.5	455.1	0.997141225

Supplemental Table S4. Effects of sodium butyrate supplementation on the taxonomic analysis of the intestinal microbiota at the phylum and genus levels in preweaning calves.

Relative abundance,%	Supplementation level, g/d				SEM	*p*-Value		
	0	4.4	8.8	17.6		ANOVA	Linear	Quadratic
Phylum level								
Firmicutes	72.187	73.529	63.716	81.499	4.7837	0.092	0.17	0.078
Actinobacteriota	10.732	7.236	7.408	9.312	2.9348	0.81	0.35	0.36
Proteobacteria	0.4086	0.9615	1.2052	0.9325	0.5014	0.72	0.27	0.34
Genus level								
Blautia	18.182	11.861	10.488	18.472	3.59	0.27	0.074	0.052
Faecalibacterium	13.60	9.24	6.24	5.34	0.77	0.30	0.19	0.39
Bacteroides	7.187	10.122	8.700	4.646	2.96	0.6	0.47	0.32
Alloprevotella	3.157	4.448	8.457	1.773	2.925	0.42	0.18	0.14
Collinsella	6.808	1.698	2.267	5.718	2.260	0.31	0.086	0.073
Unclassified_f__Lachnospiraceae	3.994	4.495	1.433	5.321	1.314	0.2	0.2	0.14
Subdoligranulum	5.112	1.068	4.518	2.624	1.3124	0.14	0.51	0.62
Norank_f__norank_o__Clostridia_UCG-014	1.449	3.698	5.516	2.2	1.609	0.31	0.065	0.063
Bifidobacterium	2.887	4.348	1.884	2.754	1.4025	0.66	0.85	0.93
Ruminococcus_torques_group	4.852	1.581	3.093	2.032	1.5724	0.47	0.37	0.51
Enterococcus	4.101	0.128	1.042	0.883	0.611	0.098	0.054	0.106
Ruminococcus_gnavus_group	0.341	0.556	0.208	0.480	0.055	0.114	0.605	0.522
Prevotella	0.804	0.753	2.342	0.419	0.361	0.241	0.154	0.124
Phascolarctobacterium	0.215	2.605	0.618	0.719	0.362	0.084	0.316	0.265
Dorea	1.138	0.234	0.305	0.685	0.174	0.24	0.055	0.065
Slackia	0.707	0.147	1.248	0.225	0.219	0.26	0.506	0.42
Tyzzerella	1.079	0.588	0.333	0.292	0.144	0.19	0.106	0.252
Butyricicoccus	0.170	1.095	0.444	0.355	0.140	0.097	0.20	0.161
Syntrophococcus	0.027	0.075	1.680	0.025	0.291	0.11	0.067	0.065

SEM = standard error of the means; $n = 10$ for each group; Means are significantly different with $p < 0.05$.

Supplemental Table S5. Effects of sodium butyrate supplementation on KEGG pathways (level 2) enriched in the intestinal microbiota in preweaning calves as analyzed using the PICRUSt program.

Relative abundance,%	Supplementation level, g/d				SEM	*p*-value		
	0	4.4	8.8	17.6		ANOVA	Linear	Quadratic
Carbohydrate metabolism	11.2887	10.7493	11.1347	11.0797	0.1890	0.25	0.55	0.51
Lipid metabolism	2.01594	2.12666	2.0929	2.11549	0.0439	0.29	0.33	0.34
Metabolism of cofactors and vitamins	3.21033	3.19308	3.1283	3.22954	0.0592	0.65	0.96	0.96
Energy metabolism	3.55297	3.52752	3.59671	3.54513	0.0402	0.66	0.77	0.65
Amino acid metabolism	5.7521	5.8266	5.7964	5.665	0.1087	0.74	0.40	0.43
Nucleotide metabolism	2.978^{b}	3.4172^{a}	3.0928^{b}	3.4892^{a}	0.0825	<0.001	0.41	0.34
Biosynthesis of other secondary metabolites	1.30781	1.29027	1.27129	1.26137	0.0285	0.67	0.32	0.34
Metabolism of terpenoids and polyketides	0.75328^{b}	0.81462^{a}	0.79701^{ab}	0.79061^{ab}	0.0151	0.048	0.61	0.51
Xenobiotics biodegradation and metabolism	1.05426^{b}	1.26039^{a}	1.2398^{a}	1.19724^{a}	0.0372	0.002	0.68	0.71
Metabolism of other amino acids	1.06295^{b}	1.14133^{a}	1.13333^{a}	1.16288^{a}	0.0234	0.027	0.44	0.43
Glycan biosynthesis and metabolism	1.24473	1.20225	1.19423	1.24645	0.0340	0.59	0.83	0.81
Translation	2.31769^{b}	2.59809^{a}	2.35064^{b}	2.65163^{a}	0.0651	0.001	0.64	0.72
Chemical structure transformation maps	1.24E-04	2.55E-04	5.53E-04	6.55E-05	0.0002	0.39	0.29	0.32
Global and overview maps	36.7537	36.5826	36.5127	36.2561	0.1859	0.31	0.76	0.79
Drug resistance: Antimicrobial	1.17203	1.22099	1.20699	1.2229	0.0286	0.57	0.90	0.94
Drug resistance: Antineoplastic	0.20869^{c}	0.23828^{ab}	0.22047^{bc}	0.25694^{a}	0.0091	0.004	0.75	0.75
Membrane transport	9.4626	8.9311	9.2064	9.2025	0.1481	0.11	0.78	0.81
Signal transduction	4.7445	4.438	4.798	4.5167	0.1881	0.47	0.11	0.12
Cellular community - prokaryotes	4.1684^{a}	3.9531^{ab}	4.1253^{a}	3.6956^{b}	0.1056	0.013	0.33	0.36
Cell motility	0.24463^{ab}	0.1959^{ab}	0.2858^{a}	0.16215^{b}	0.0319	0.048	0.76	0.75
Folding, sorting and degradation	1.23432	1.29694	1.22712	1.2988	0.0309	0.21	0.32	0.37
Transcription	0.151819^{b}	0.179222^{a}	0.158838^{b}	0.193429^{a}	0.0061	<.0001	0.43	0.43
Replication and repair	2.14779^{b}	2.42651^{a}	2.20949^{b}	2.5207^{a}	0.0532	<0.001	0.86	0.89
Endocrine system	0.42943^{b}	0.50287^{a}	0.44593^{b}	0.4882^{a}	0.0146	<0.001	0.33	0.36
Signaling molecules and interaction	0.00158	0.000302	0.00127	0.0002	0.0005	0.12	0.49	0.56
Cell growth and death	0.75388^{a}	0.69414^{ab}	0.70409^{ab}	0.64226^{b}	0.0214	0.008	0.77	0.81
Transport and catabolism	0.2103^{a}	0.1732^{ab}	0.18377^{a}	0.14591^{b}	0.0124	0.008	0.26	0.81
Aging	0.195262	0.209379	0.19834	0.210167	0.0058	0.18	0.65	0.93
Circulatory system	0.005438^{c}	0.023001^{a}	0.014762^{b}	0.028521^{a}	0.0028	<0.001	0.06	0.47

Development	0.00019161	0.00005114	0.00014271	0.0000115	0.0001	0.27	0.64	0.95
Cellular community - eukaryotes								
Immune system	0.131534^{a}	0.109318^{bc}	0.12273^{ab}	0.105969^{c}	0.0048	0.002	0.23	0.63
Environmental adaptation	0.104089	0.104745	0.099976	0.088539	0.0047	0.074	0.96	0.42
Nervous system	0.13011^{b}	0.144048^{a}	0.132623^{ab}	0.144021^{a}	0.0045	0.057	0.60	0.91
Endocrine and metabolic diseases	0.163618	0.171668	0.163524	0.165829	0.0038	0.41	0.71	0.68
Excretory system	0.01666^{c}	0.052966^{a}	0.036332^{b}	0.063074^{a}	0.0054	<0.001	0.043	0.37
Digestive system	0.05735^{b}	0.11933^{a}	0.08057^{b}	0.12429^{a}	0.0121	<0.001	0.20	0.56
Neurodegenerative diseases	0.03678	0.04318	0.046634	0.036372	0.0039	0.2	0.048	0.033
Substance dependence	0.00822	0.011831	0.007427	0.006385	0.0036	0.73	0.85	0.70
Infectious diseases: Bacterial	0.37994	0.40212	0.40442	0.3886	0.0092	0.21	0.04	0.04
Infectious diseases: Parasitic	0.007624	0.011739	0.009256	0.008585	0.0026	0.72	0.48	0.45
Infectious diseases: Viral	0.07194	0.06127	0.05768	0.04358	0.0091	0.19	0.46	0.89
Cancers: Overview	0.28843^{b}	0.3475^{a}	0.31669^{ab}	0.34781^{a}	0.0108	<0.001	0.078	0.28
Cancers: Specific types	0.04494^{b}	0.0588^{a}	0.054305^{ab}	0.054208^{ab}	0.0028	0.011	0.053	0.057
Immune diseases	0.02891^{c}	0.039889^{ab}	0.031927^{bc}	0.046504^{a}	0.0034	0.003	0.69	0.66
Cardiovascular diseases	0.106367	0.108545	0.107697	0.100814	0.0061	0.81	0.70	0.53

SEM = standard error of the means; n = 10 for each group; Means within row with the different lowercase letters superscripts are significantly different ($p < 0.05$). "—": not detected.

Supplemental Table S6. Relationship of growth-related indices of calves and indices of the intestinal microbiota.

Items	*Bacteroidota*	*Lactobacillus*	*Norank_f__Muribaculaceae*	*Rikenellaceae_RC9_gut_group*	*Norank_f__Eubacterium_coprostanoligenes_group*	*Lachnoclostridium*	*Ruminococcus*	*Romboutsia*	*Lachnospiraceae_NK4A136_group*	*Erysipelotrichaceae_UCG-003*	*Olsenella*	Shannon index
BW	0.330*	0.142	0.318*	0.299	0.098	–0.135	0.267	–0.088	0.089	0.040	0.050	0.367*
ADG	0.234	–0.164	0.291	0.295	0.204	0.020	0.345*	0.072	0.306	–0.197	0.192	0.577*
FDMI	0.290	–0.552*	0.183	0.402*	0.199	0.307	0.080	–0.113	–0.142	–0.388*	–0.012	–0.127
FE	0.218	–0.299	–0.041	0.008	0.154	0.087	–0.056	–0.029	–0.201	–0.208	–0.026	-0.026

* $p < 0.05$; BW: body weight; ADG: average daily gain; DMI: dry matter intake; FE: feed efficiency. The correlation using Spearman's rank correlation coefficient and 2–tailed significance test ($n = 40$).

A

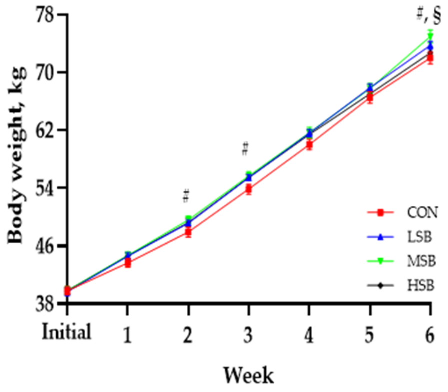
Body weight, kg
78
70
62
54
46
38
Initial
1
2
3
4
5
6
Week
#
#
#, §
CON
LSB
MSB
HSB

B

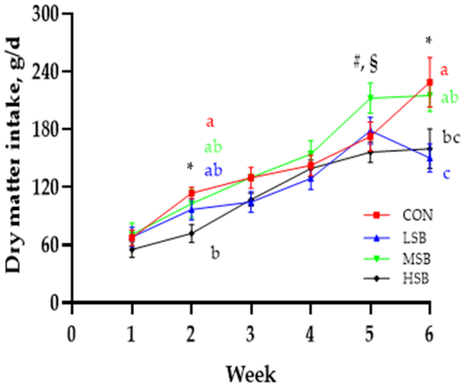
Dry matter intake, g/d
300
240
180
120
60
0
0
1
2
3
4
5
6
Week
*
a
ab
ab
b
#, §
*
a
ab
bc
c
CON
LSB
MSB
HSB

C

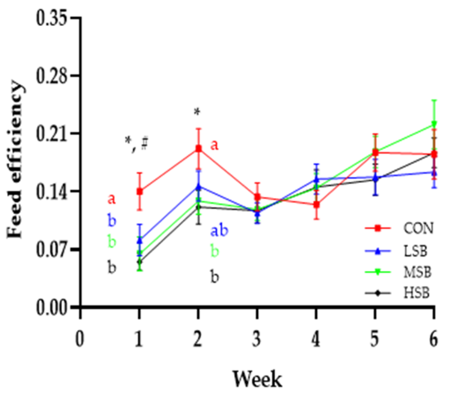
Feed efficiency
0.35
0.28
0.21
0.14
0.07
0.00
0
1
2
3
4
5
6
Week
*, #
a
b
b
b
*
a
ab
b
b
CON
LSB
MSB
HSB

D

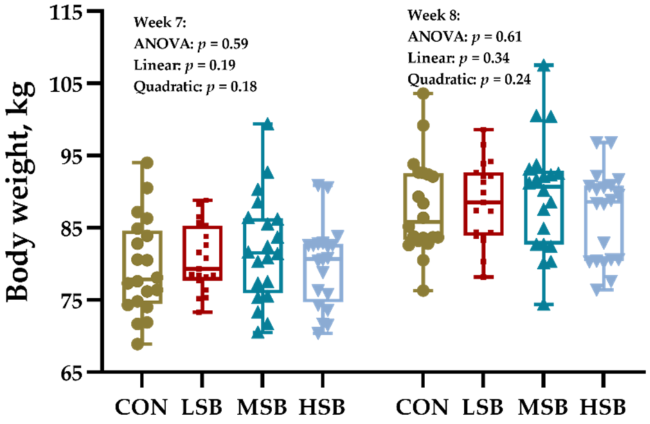

E

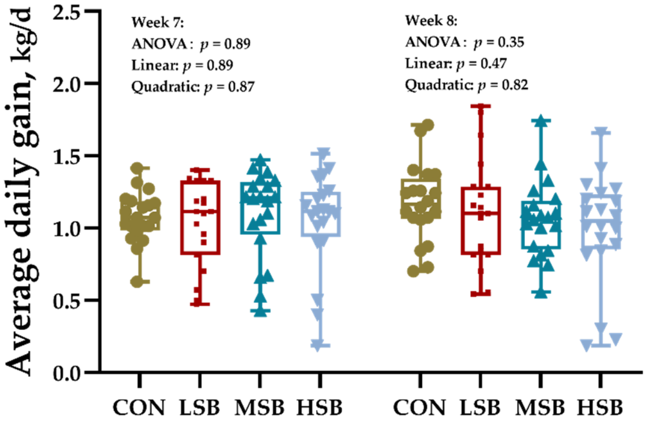

Supplemental Figure S1. Growth performance of preweaning calves in each week and the body weight and average daily gain of calves at 46–59 d of age (weeks 7 and 8; weaning transition period). CON = treatment consisted of milk without SB supplementation for calves (0 g/d), LSB = treatment consisted of milk with a low level of SB supplementation for calves (4.4 g/d), MSB = treatment consisted of milk with a medium level of SB supplementation for calves (8.8 g/d), and HSB = treatment consisted of milk with a high level of SB supplementation for calves (17.6 g/d). All the results are shown as the mean ± SEM, n = 20 (n = 19 for the LSB group at week 4 and thereafter). FE was calculated by DMI/ADG. The four treatments are shown with ANOVA (* $p < 0.05$; ** $p < 0.01$), linear (# $p < 0.05$; ## $p < 0.01$), and quadratic effects (§ $p < 0.05$; §§ $p < 0.01$) at the same time period (week), and different lowercase letters for different treatments at the same time period are shown when significantly different ($p < 0.05$).

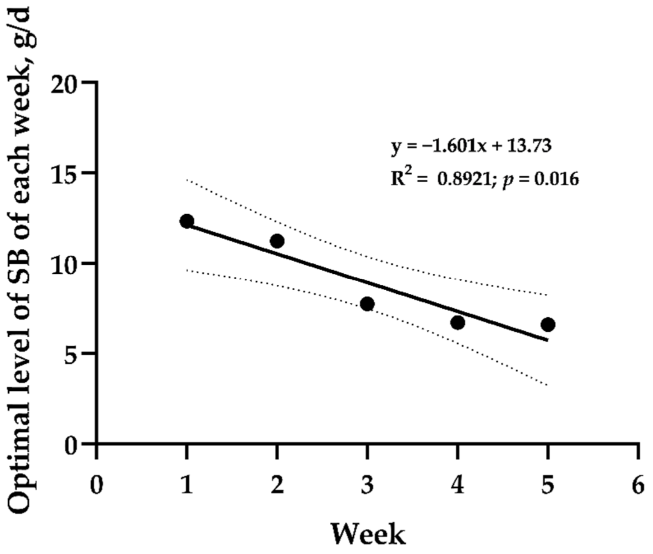

Figure S2. Result of regression analysis for the obtained optimal sodium butyrate supplementation level for each week. CON = treatment consisted of milk without SB supplementation for calves (0 g/d), LSB = treatment consisted of milk with a low level of SB supplementation for calves (4.4 g/d), MSB = treatment consisted of milk with a medium level of SB supplementation for calves (8.8 g/d), and HSB = treatment consisted of milk with a high level of SB supplementation for calves (17.6 g/d). Using second-order polynomial regression (quadratic) equations on ADG for each week, and the optimal SB supplementation level were 12.33, 11.24, 7.74, 6.71, and 6.59 for 1st, 2nd, 3rd, 4th, and 5th weeks.

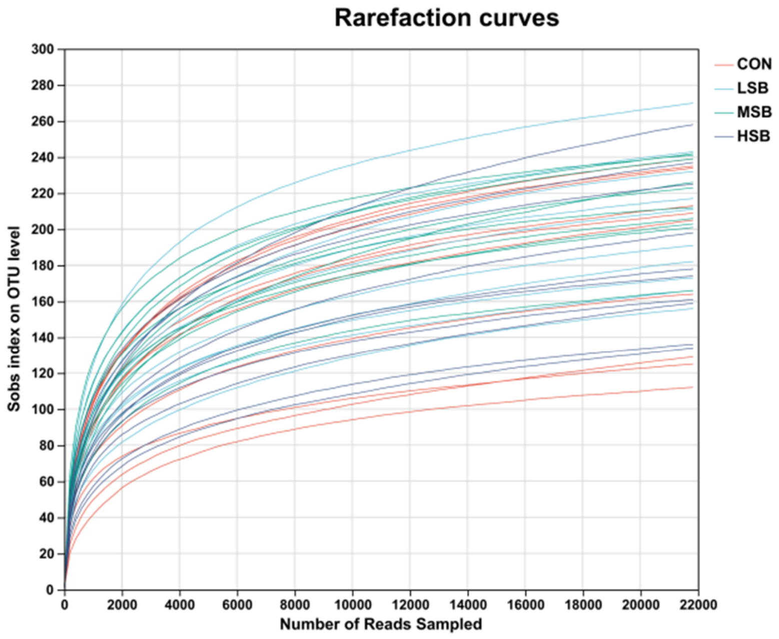

Figure S3. Effects of sodium butyrate supplementation on rarefaction curves of the intestinal microbiota in preweaning calves. CON = treatment consisted of milk without SB supplementation for calves (0 g/d), LSB = treatment consisted of milk with a low level of SB supplementation for calves (4.4 g/d), MSB = treatment consisted of milk with a medium level of SB supplementation for calves (8.8 g/d), and HSB = treatment consisted of milk with a high level of SB supplementation for calves (17.6 g/d).

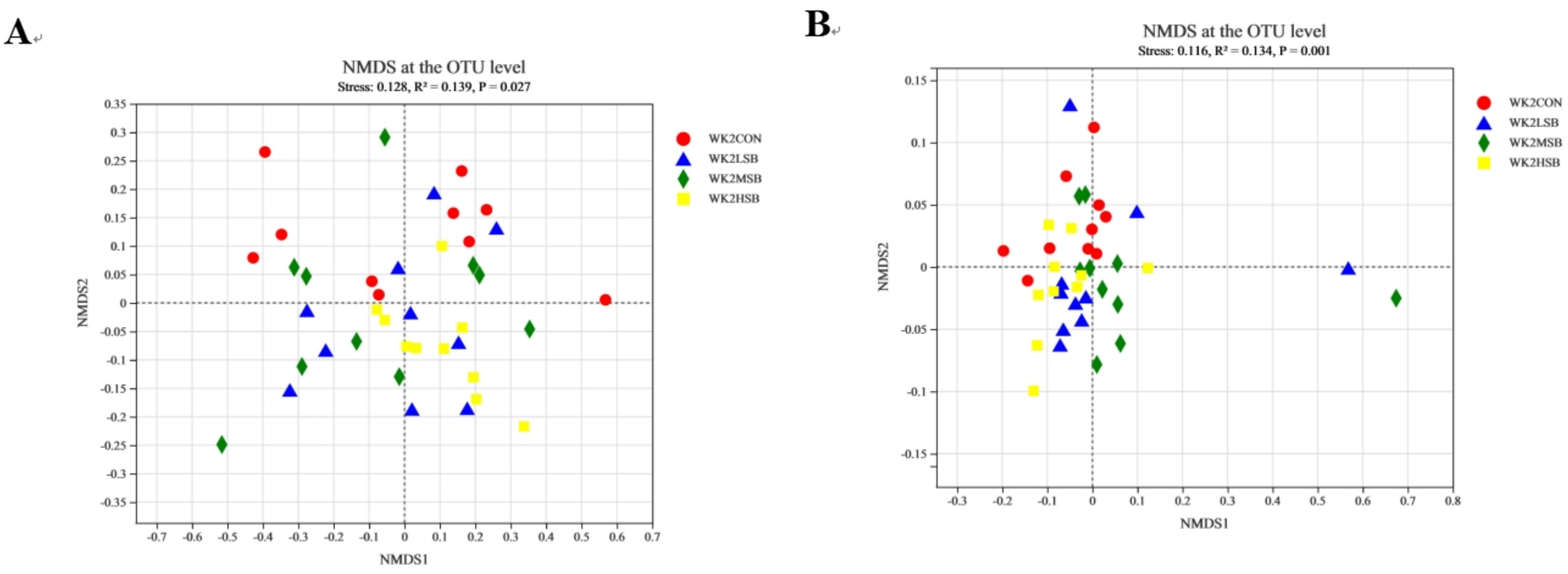

Figure S4. Effects of sodium butyrate supplementation on beta diversity of the intestinal microbiota in preweaning calves. Beta diversity measures using NMDS for the intestinal microbiota communities and NMDS plot based on community membership as measured by the weighted UniFrac distances (**A**) and unweighted UniFrac distances (**B**) followed by the Adonis test at the OTU level. CON = treatment consisted of milk without SB supplementation for calves (0 g/d), LSB = treatment consisted of milk with a low level of SB supplementation for calves (4.4 g/d), MSB = treatment consisted of milk with a medium level of SB supplementation for calves (8.8 g/d), and HSB = treatment consisted of milk with a high level of SB supplementation for calves (17.6 g/d). *n* = 10 for each group.

A

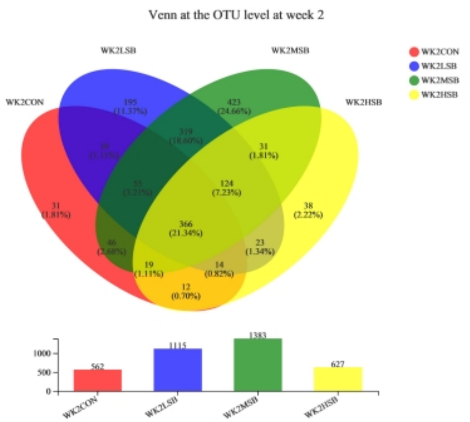

B

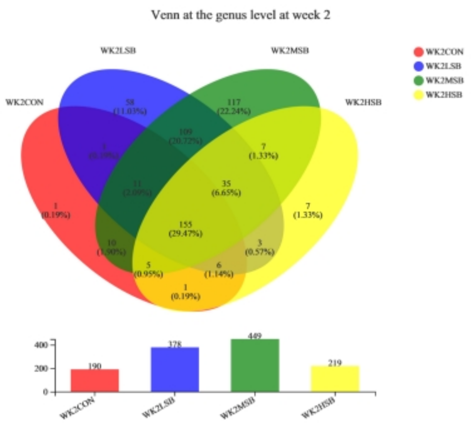

C

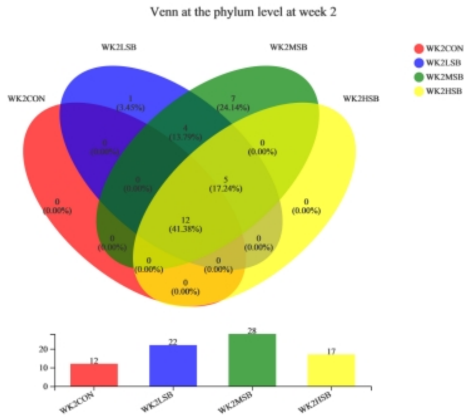

Figure S5. Venn diagram of the common and distinct taxa at the phylum, genus, and OTU levels among the four groups of preweaning calves. Venn's diagram at the OTU (**A**), genus (**B**), and phylum (**C**) levels. CON = treatment consisted of milk without SB supplementation for calves (0 g/d), LSB = treatment consisted of milk with a low level of SB supplementation for calves (4.4 g/d), MSB = treatment consisted of milk with a medium level of SB supplementation for calves (8.8 g/d), and HSB = treatment consisted of milk with a high level of SB supplementation for calves (17.6 g/d). *n* = 10 for each group.
